# Generative Data Augmentation for ArUco-Free RGB-Based 6-DoF Object Pose Estimation

**DOI:** 10.3390/jimaging12060244

**Published:** 2026-05-29

**Authors:** Carmelo Scribano, Iacopo Ferrari, Giorgia Franchini, Elena Govi, Davide Sapienza, Tobia Poppi, Micaela Verucchi, Marko Bertogna

**Affiliations:** 1 Department of Physics, Informatics and Mathematics, University of Modena and Reggio Emilia, Via Campi 213/B, 41125 Modena, Italy; carmelo.scribano@unimore.it (C.S.); davide.sapienza@unimore.it (D.S.); tobia.poppi@unimore.it (T.P.); marko.bertogna@unimore.it (M.B.); 2 HIPERT s.r.l, Via Inventori, 37, 41121 Modena, Italy; elena.govi@hipert.it (E.G.); micaela.verucchi@hipert.it (M.V.)

**Keywords:** data manipulation, 6d pose estimation, generative data augmentation, explainable artificial intelligence, saliency methods

## Abstract

In recent years, data-driven approaches have become increasingly important in industrial computer vision applications, particularly for 6-Degrees-of-Freedom (6-DoF) object pose estimation. However, benchmark datasets may unintentionally introduce biases that affect the reliability of learned models. In this work, we investigate the shortcut bias induced by fiducial ArUco markers in the widely used Linemod dataset. Although such markers are typically absent in real industrial environments, they introduce unintended visual cues that neural networks tend to exploit. As a result, model selection based on state-of-the-art benchmarks can be biased, since the reported performance often reflects reliance on these shortcuts rather than on robust feature extraction. Using saliency map analysis, we show that often a large portion of the model’s attention is concentrated on these markers, revealing the presence of a shortcut that artificially boosts pose estimation performance. To mitigate this issue, we propose a data augmentation pipeline based on generative AI techniques that removes the markers and replaces the background with more realistic synthesized scenes. Experimental results indicate a noticeable drop in performance when models trained on the original Linemod dataset are evaluated in ArUco-free environments, confirming the presence of background-induced biases. Training with the proposed generative-swapped dataset leads to improved robustness and better generalization to unseen scenarios, although it does not fully eliminate the problem. Overall, the results highlight the impact of background-related biases in pose estimation benchmarks and suggest that the proposed augmentation strategy represents a practical and scalable step toward developing more reliable 6-DoF pose estimation systems for industrial applications, while leaving room for further improvements.

## 1. Introduction

### 1.1. Domain of Interest

Recovering the 6-Degrees-of-Freedom (often referred to as 6D or 6-DoF) pose of an object from a single Red Green Blue (RGB) image is a relevant computer vision problem with applications in industrial automation [1], robotics [2,3], automotive [4,5], augmented reality [6] and beyond.

Given an RGB image $I\in R^{h\times w\times 3}$, a 6-DoF pose estimation algorithm aims to recover the translation $t=(t_{x},t_{y},t_{z})$ and rotation $R=(r_{x},r_{y},r_{z})$ vectors that define the position and orientation of an object in the camera coordinate system [7]. Recent learning-based techniques, particularly those based on Deep Neural Networks (DNNs), achieve state-of-the-art performances in 6-DoF object pose estimation. Convolutional Neural Networks (CNNs), in particular, achieved impressive results in a wide variety of computer vision problems, including 6-DoF pose estimation from RGB input, at the cost of being extremely data-driven and requiring significant amounts of labeled training examples. In Section 2.1, we present a taxonomy of the most popular Deep Learning methods for 6-DoF object pose estimation. For other computer vision tasks the dataset acquisition and labeling are straightforward to obtain, utilizing manual labeling; however, this is not the case for 6-DoF object pose, since identifying ground truth translations and rotations from real images is not easily feasible for a human annotator. The research community has resorted to either relying on large datasets obtained from a photorealistic simulation [8] or to smaller datasets of real-world images labeled by relying on fiduciary markers [9,10].

### 1.2. Annotation-Induced BIAS

**Figure 1:**
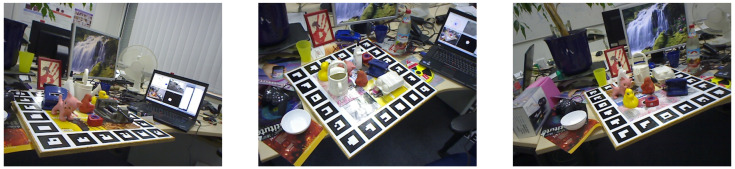
Samples from the Linemod dataset.

We refer the reader to Section 2.1 for an overview of the datasets present in the literature. We are particularly interested in real-world datasets that rely on markers to extract the object pose ground truth. It is well known, as reported in the literature [11], that data-driven models, and deep models in particular, can learn unintended or spurious correlations from the training data, often referred to as “shortcut biases”. This results in accurate predictions on the benchmark dataset, while causing failure on real-world examples where the learned correlations do not hold. We investigate whether the presence of easily detectable fiducial markers introduces such shortcut biases in 6-DoF pose estimation. In particular, we focus on the widely used Linemod (LM) dataset [9], shown in Figure 1. In this dataset, objects are placed on a board surrounded by Augmented Reality University of Cordoba (ArUco) markers [12]. The ground truth pose is obtained by estimating the board pose via marker detection and applying a fixed transformation to the object.

Since the markers and the board are visible in the training and evaluation images, models may exploit these cues instead of learning intrinsic object geometry. Additionally, the fixed arrangement of the objects on the board could further reinforce unintended correlations. As LM is a single-object dataset, only the pose of the central object is provided, while other objects remain visible. This setup raises the possibility that models leverage contextual information from surrounding objects to infer the target pose.

These observations raise important questions regarding generalization:

- Does the network exploit markers and surrounding objects as shortcuts for pose estimation?
- To what extent does object placement bias the learning process?
- To what extent is the method affected by a static or semi-static background?

### 1.3. Methodology Overview

We propose a two-stage methodology that combines diagnostic analysis with generative data augmentation. First, we investigate the presence of shortcut biases using visual interpretability techniques, specifically Gradient-weighted Class Activation Mapping (Grad-CAM). This allows us to analyze how models trained on LM distribute attention operate and whether they rely on fiducial markers rather than object features. Second, we introduce a generative data augmentation strategy to mitigate these biases. We construct an ArUco-Free (AF) version of the dataset by removing ArUco markers through generative inpainting using a state-of-the-art generative model. We investigate whether a similar process can preserve the object geometry while removing spurious contextual cues and encourage the model to focus on intrinsic object features.

#### 1.3.1. Contributions

The main contributions of this work are summarized as follows:

- We assess and analyze the presence of shortcut biases learned from 6-DoF pose estimation models. In particular, we investigate the shortcut arising from fiducial markers in a popular training dataset.
- We propose a generalized Grad-CAM formulation for regression tasks, enabling the visualization of sensitivity maps for 6-DoF pose estimation models.
- We introduce an ArUco-free (AF) version of the LineMod dataset, obtained via generative inpainting, to remove marker-induced biases while preserving scene realism.
- We provide a comprehensive experimental evaluation demonstrating the impact of annotation-induced bias on model performance and generalization, highlighting the trade-off between accuracy and robustness.

#### 1.3.2. Paper Organization

In Section 2, the state-of-the-art for 6-DoF pose estimation datasets is described, providing insights into the 6-DoF pose Deep Learning methods, with a focus on EfficientPose (EP). Additionally, the Saliency maps methods for interpreting the learning process are introduced. In Section 3, experiments we have performed to demonstrate our hypothesisare are described in detail. In Section 4, the numerical and visual results are discussed, with their limitations and consequences. Finally, in Section 5, concluding coments are provided.

## 2. Related Works

### 2.1. 6-DoF Pose Estimation Datasets

#### 2.1.1. Linemod and Linemod-Occluded

**Figure 2:**
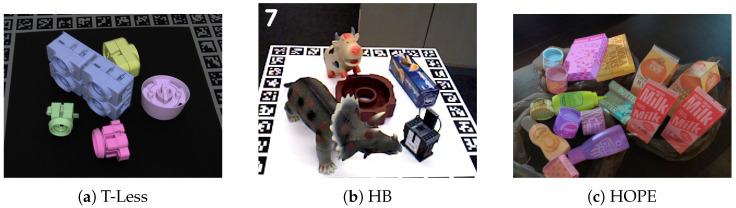
Sample images from popular 6DoF pose estimation datasets.

Linemod [9] is one of the most widely adopted benchmarks in the 6-DoF pose estimation domain; it consists of real images containing 15 object classes, captured from multiple viewpoints. For each object class, a subset of images is provided with ground-truth 6-DoF pose annotations for the target object only. In these images, the target object is typically placed near the center of a custom-made working plane and surrounded by clutter that induces only mild occlusion. As previously noted, the working plane features a chessboard-like structure with embedded ArUco markers, which are leveraged for the ground-truth pose annotation. Building on this dataset, Linemod-Occluded (LM-O) [10], provides additional ground-truth annotations for all objects of a subset of the LM images (the bench vise object subset), allowing for training and testing of multi-object pose estimation methods. These scenes include significant inter-object occlusions, making the task substantially more challenging (see Figure 2). LineMod exhibits several limitations, including constrained object placement, limited background variability, relatively simple lighting conditions, and the clear presence of fiducial markers. Nevertheless, it remains relevant and widely adopted in the 6-DoF pose estimation literature; its long-standing adoption has established it as a standard reference point, facilitating consistent comparison across methods. Additionally, its simplicity allows for validating methodologies in a controlled setup before moving to more complex and unconstrained scenarios.

#### 2.1.2. T-LESS

The T-LESS dataset, introduced in [8], consists of 30 industry-relevant objects which lack texture or discernible color, as well as 20 Red Green Blue-Depth (RGB-D) scenes that were recorded through three synchronized cameras: the PrimeSense Carmine 1.09, the Kinect 2 RGB-D cameras, and the Canon RGB camera. The objects featured in the dataset present some symmetries and mutual similarities, with some being a combination of multiple objects. The ground-truth poses in T-LESS are derived from a calibrated turntable setup integrated with a fiducial marker field to guarantee high geometric precision. Such a methodology underscores a common trend: even benchmarks targeting texture-less objects frequently depend on markers for accurate ground-truth generation, which may introduce the same types of contextual shortcuts observed in LM.

#### 2.1.3. HomebrewedDB

Structurally similar to T-LESS, HomebrewedDB (HB) [13] covers a wider range of objects and provides more challenging occlusions. It consists of 33 highly accurate 3D models of toys, household objects, and low-textured industrial objects of varying sizes, along with 13 sequences containing 1340 frames filmed with two RGB-D sensors. The scenes range from simple (three objects on a plain background) to complex (highly occluded with 8 objects and extensive clutter). Similar to LineMod, HB utilizes an ArUco markerboard to automate camera tracking and ensure a precise ground truth. This confirms that marker-aided acquisition is a widespread practice across benchmarks, reinforcing the need to investigate how these patterns influence model learning.

#### 2.1.4. HOPE

NVIDIA Houseold Objects for Pose Estimation (HOPE) [14] introduced a new dataset of toy grocery objects. The annotations for this dataset were obtained manually, through the identification of point correspondences between images and 3D textured object models. During the acquisition phase, ten different environments, with 5 object arrangements/camera poses per environment, were used. These 50 different scenes exhibit a wide variety of backgrounds, clutter, poses, and lighting. To provide additional clutter and partial occlusion, objects are also placed in other containers, such as bags or boxes. Of significance in the scope of this paper, the dataset is advantageous as it does not utilize markers or ArUco markers during acquisition. Moreover, the different environments permit better generalization. In total, the dataset contains 50 unique scenes, 238 images, and 914 object poses. Once the camera and the object position are known, some light effects are applied in order to have more images with little differences in shadows and change colors, thereby resulting in more static images that do not need to be annotated. HOPE adopts a different strategy by utilizing a semi-manual annotation process based on 2D–3D point correspondences, avoiding markers entirely. While this approach successfully eliminates marker-induced shortcuts, it leads to an estimated ground truth rather than a purely geometric one, highlighting the inherent trade-off between ensuring an unbiased environment and achieving absolute pose accuracy.

### 2.2. Generative Models

**Figure 3:**
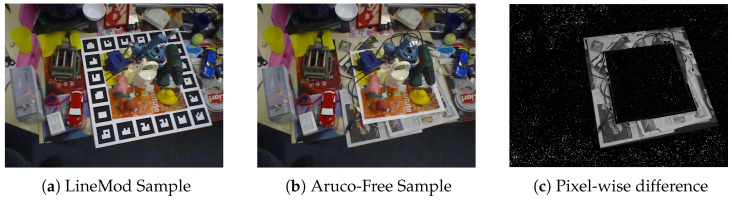
Example of marker removal using generative inpainting.

Generative models have recently emerged as powerful tools for synthesizing realistic visual data and augmenting existing datasets in computer vision applications. Unlike simulation-based pipelines, which generate synthetic images using explicit 3D rendering engines and physical models, generative approaches learn the underlying distribution of real data directly from samples. This allows the creation of realistic images that preserve the statistical properties of real-world observations while introducing controlled variations in appearance and context. Early deep generative approaches include Variational Autoencoders (VAEs) [15] and Generative Adversarial Networks (GANs) [16]. In particular, GAN-based architectures have demonstrated strong capabilities in image synthesis tasks, making them suitable for data augmentation when labeled data are scarce or expensive to acquire. Several works have explored GAN-based augmentation techniques to improve the robustness and generalization of deep learning models in visual recognition tasks [17,18]. More recently, diffusion-based generative models [19,20] have significantly improved the realism and diversity of synthesized images, enabling the creation of high-quality datasets that closely resemble real-world distributions. Generative data augmentation has also been explored in the context of pose estimation and 3D perception. For example, synthetic data generation has been widely used to train pose estimation systems when real annotated datasets are limited [2]. In addition, domain adaptation approaches such as the Simulation Generative Adversarial Network (SimGAN) [18] have been proposed to bridge the gap between synthetic and real data by refining simulated images to make them appear more realistic. More recent works combine generative models with data augmentation pipelines to create diversified training datasets that improve the robustness of object detection and pose estimation models under varying backgrounds, illumination conditions, and occlusions. In contrast to simulation-based dataset generation, which relies on rendering objects in virtual environments, generative approaches operate directly in the image domain and modify existing images while preserving their semantic structure. This makes them particularly suitable for addressing dataset biases that arise from unintended correlations between objects and background features. In the context of pose estimation benchmarks, such biases may lead neural networks to exploit spurious cues rather than learning meaningful geometric representations. Motivated by these observations, in this work, we adopt a generative data augmentation strategy to mitigate the shortcut bias introduced by fiducial markers in the LM dataset. We formulate the removal of ArUco markers as a generative inpainting and scene-composition task. By leveraging latent diffusion architectures, we perform a semantic-aware restoration of the occluded regions, effectively decoupling the object’s geometric features from the spurious cues provided by the markers. This process does not merely erase the fiducial patterns but re-synthesizes the underlying environment, ensuring that the resulting training samples remain distributionally consistent with real-world, marker-less scenarios (see Figure 3). To this end, we leverage a state-of-the-art commercial solution and qualitatively evaluate whether the image-to-image generation has only affected the region of the image where markers are visible. The complete details of the dataset generation are discussed in Section 3.1.

### 2.3. 6-DoF Pose Estimation Models

6-DoF pose estimation consists of predicting the rotation and translation of an object. This task is crucial because it determines the exact spatial location and orientation of objects, making 6-DoF object pose estimation increasingly vital for a wide range of computer vision applications. As computer vision theory advances and related fields rapidly evolve, research into 6-DoF object pose estimation has expanded significantly, offering deeper insights and driving innovation in the field. The objective of 6-DoF object pose estimation is to approximate a function *f* that maps an RGB input image $I\in R^{h\times w\times 3}$ to a pose P in the special Euclidean group SE(3):

(1) $$f:I\in R^{h\times w\times 3}\to P\in SE\left(3\right)$$

Based on the availability of the 3D object model (i.e, Polygon mesh) M during training and testing, the literature typically distinguishes between *Model-Based* and *Model-Free* approaches, categorized as follows:

- Instance-Level Object Pose Estimation (Model-Based): A specific set of object models $M=\{M_{0},\ldots ,M_{m}\}$ is provided. The model $M_{i}$ for each object $O_{i}$ is known during both training and inference, making this approach suitable for *seen* objects.
- Category-Level Object Pose Estimation (Model-Based): The exact model $M_{i_{0}}$ is not provided. Instead, the estimator is trained on similar models $\left\{M_{i_{1}},\ldots ,M_{i_{s}}\right\}$ belonging to the same category, allowing it to build prior knowledge for *unseen* instances of a known class. Examples include [21,22,23].
- Zero-Shot Novel Object Pose Estimation (Model-Free): The estimator is entirely model-free and is required to predict the pose of *novel* objects without any prior geometric or categorical knowledge [24,25].

In this paper, we will focus on the specific category of RGB-Input, Instance-Level Object Pose Estimation. Current RGB-based estimators can be broadly categorized into three main tactical groups based on their pipeline architecture:

- Direct Regression Methods: These approaches directly map the input image or Region of Interest (RoI) to 6-DoF pose parameters (rotation and translation) in an end-to-end fashion. They can be single-stage [26,27,28] or multi-stage systems that first perform 2D detection before regressing the pose [29,30].
- Correspondence-Based Methods (2D–3D): These methods establish spatial relationships between 2D image pixels and 3D object coordinates. The final pose is recovered either via deterministic algorithms like Perspective-n-Point (PnP) with RANSAC (Random Sample Consensus) [31,32,33,34,35] or through differentiable PnP layers that allow for end-to-end training [36,37,38].
- Template and Latent Matching: Drawing from traditional geometric feature matching [39,40], modern variants use deep learning to perform template matching in a discretized orientation space [41] or leverage autoencoders to learn implicit latent representations of object rotations [42].

#### EfficientPose

EfficientPose (EP) [27] is an extension of a widely used 2D detector, EP [43], based on the popular convolutional backbone EfficientNet [44]. In a single-stage, the architecture is able to predict the class, the 2D bounding box, rotation, and translation of one or more objects, given an RGB image as input. In detail, two analogous to the classification and bounding box regression subnetworks are added to the Encoder-Decoder (ED), modeled after the classification and bounding box regression of the original model. The rotation subnetwork predicts the rotation vector $r\in R^{3}$, in an axis-angle representation. Its architecture is similar to the class and bounding box regression, with the addition of an iterative refinement module. The final rotation is then the sum $r=r_{init}+\Delta r$ where $r_{init}$ is the initial estimate for the rotation, while $\Delta_{r}$ is the iterative refinement module, given as the output of separable convolutional layers, group normalization, and activation functions. The translation network on the other side shares a similar structure. Instead of regressing directly $\left(t_{x},t_{y},t_{z}\right)$, the t-architecture predicts separately $\left(c_{x},c_{y}\right)$, which represents the object center in the image, and $t_{z}$. After this, $t_{x}$ and $t_{y}$ are obtained from $\left(c_{x},c_{y}\right)$ and fixed camera parameters, as reported in [29]. To maintain the scalability of the base architecture, EP utilizes a global scaling hyperparameter ϕ∈{1,3}. This parameter simultaneously controls the depth and width of the backbone, and the resolution of the input image, as well as the complexity of the pose subnetworks. In this work, we employ the ϕ=0 configuration, which represents the most computationally efficient version of the model. We refer the reader to the original publication [27] for the detailed scaling equations.

### 2.4. Saliency Maps: Explanation

**Figure 4:**
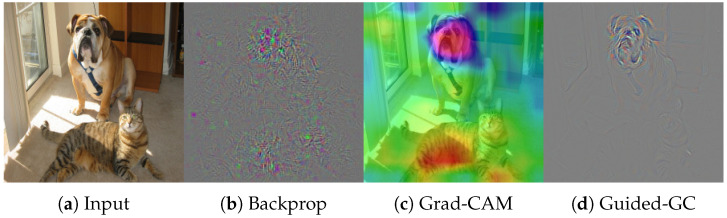
Output of saliency methods for the class Mastiff. Source [48].

Interpretable Machine Learning (IML) [45] has experienced rapid growth in the Machine Learning domain, primarily focusing on elucidating the process behind a model’s specific prediction. While some models, such as Decision Trees and Rule-Based Classifiers, are inherently interpretable, DNNs cannot be interpreted in the same manner and are generally considered to be black-box predictors after training. Pixel attribution is a family of attribution methods designed to interpret the prediction of models that operate on images, with the aim of producing a saliency map that highlights the importance of individual (or groups of) pixels in the input image for the model’s specific output. One of the simple methods to generate saliency maps, introduced in [46], is the Guided Backpropagation (sometimes referred to as Vanilla-Gradient), and it consists of computing the gradient of the output prediction with respect to the input image (an example is given in Figure 4). More formally, given s=Ψ(I), where Ψ(·) denotes the (trained) neural network, the gradient $\frac{\partial \Psi }{\partial I}$ can be easily obtained via standard backpropagation. Grad-CAM [47] is a more recent method to produce saliency maps: differently from the vanilla gradient, the gradient of the output $y_{s}$, that is the class score before the softmax, is computed with respect to the output feature map $A^{k}$ of a convolutional layer (ideally, the last one before the global average pooling of traditional classification models). The gradient tensor is then averaged across the spatial dimensions indexed by i,j to obtain a weight vector $\alpha^{s}=\left[\alpha_{1}^{s},\alpha_{2}^{s},...,\alpha_{c}^{s}\right]\in R^{c}$, where each element $a_{k}^{s}$ is computed as:

(2) $$\alpha_{k}^{s}=\frac{1}{Z}\sum_{i}\sum_{j}\frac{\partial y_{s}}{\partial A_{ij}^{k}}$$

Finally, the saliency maps are obtained by weighting each channel of the feature map $A^{k}$ with the corresponding value of $\alpha_{k}$:

(3) $$L_{GC}=ReLU\left(\sum_{k}a_{k}A^{k}\right)$$

We refer the interested reader to the exceptional work of [45] for a deeper understanding of the discussed methods and several others, which are beyond the scope of this work. In Section 3.2, we will discuss our generalization of Grad-CAM for the regression problem of our interest.

## 3. Methodology

This research aims to explore the potential for bias to be introduced into the model due to the presence of visible artifacts, particularly markers employed in the data collection process. We will focus our experimentation on the LM dataset, a well-known non-synthetic dataset, and the EP model, which is currently the most effective fully convolutional network for LM. We propose a mixed evaluation of qualitative observations, utilizing the attribution methods discussed in Section 2.4, and quantitative experimentation, involving the evaluation of results obtained from modified versions of the LM dataset, to assess the validity of our hypothesis.

The proposed methodology can be briefly summarized as follows:

- Leveraging state-of-the-art generative models, we produce an augmented version of LM by inpainting the visible markers with a realistically synthesized background. The 6-DoF pose estimation task is then compared between the augmented and the original training of EP.
- In addition, we introduce a generalization of pixel attribution methods for a regression problem, and show that the saliency maps produced with the extended version of Grad-CAM support our hypothesis that the model’s predictions are contingent upon the presence of fiduciary markers on Linemod.

In the remainder of this section, we detail the building blocks of the proposed methodology; then in Section 4, the conducted analysis is proposed and discussed in detail.

### 3.1. ArUco-Free Dataset Generation

Generating the ArUco-Free (AF) dataset requires preserving a direct correspondence between the original environment and its version without artificial landmarks. To achieve this, we developed a semi-automated pipeline leveraging state-of-the-art generative inpainting techniques. The inpainting process is orchestrated via API calls to the Nano Banana 2 generative engine (Gemini 3.1 Flash Image) (Date API accessed: 9 December 2025). The model was specifically instructed with the identification and removal of ArUco markers with a structured prompt aimed at preserving the scene layout:

*“Change the ArUco markers to a cluttered background. Generate an image in which you have modified the ArUco Markers by replacing them with consistent objects from the rest of the background. Keep all other objects exactly in their original positions.”*

**Figure 5:**
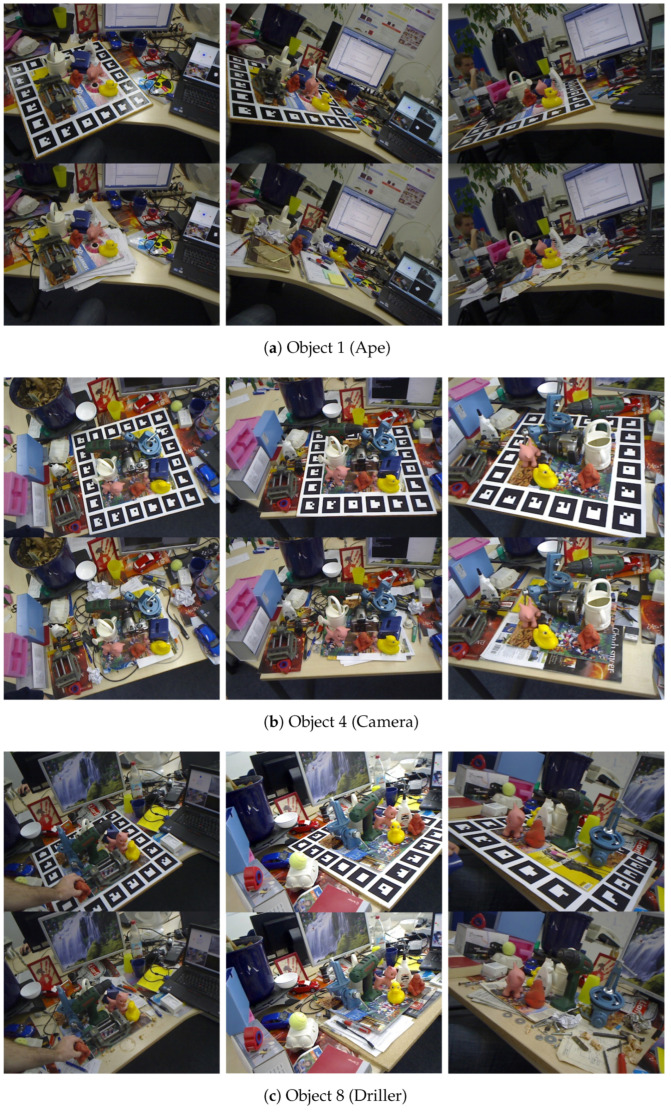
Comparison between the original LineMod images and the ArUco-Free images.

As illustrated in Figure 5, rather than performing a simple texture fill or blurring, the generative model synthesized context-aware objects (e.g., small tools, surface imperfections, or cables) that matched the semantic complexity of the surrounding environment. To enforce photometric consistency between the source and the synthesized data, the images underwent a specific post-processing normalization workflow:

- Rescaling and Alignment: LineMod images came at a resolution of 640×480, which implies a 4:3 aspect ratio. The exact resolution is not supported as Gemini 3.1 output resolution. Hence, we requested a 4:3 aspect ratio, which produced a 1200×896 upscaled image. After rescaling, a sub-pixel alignment check was performed to ensure a perfect one-to-one spatial mapping between the pixels of the original and the modified frames.
- Pixel-wise Consistency Validation: Although the model autonomously identified the markers, as shown in Figure 3c, we performed a pixel-wise comparison between the original and the generated frames to ensure that the generative process was strictly confined to the regions of interest. This validation step is presented in detail in the following paragraph.

#### Evaluation

**Figure 6:**
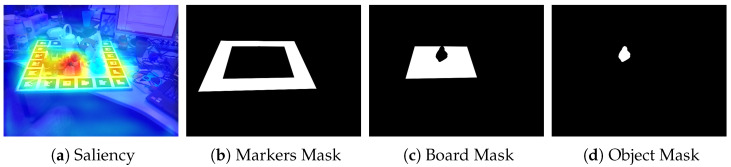
Distribution of the ArUco Board in LineMod Images for different object sets.

Finally, to assess the quality of the inpainting, we measure the pixel-wise difference between the original frame (containing markers) and the synthesized frame (without markers). Table 1 reports the mean pixel difference within the marker regions, defined by the binary mask of the ArUco marker region (see Figure 6), and over the remaining image area. The results indicate a substantial difference within the marker regions, while changes in the rest of the image remain minimal, as illustrated in Figure 3. This simple experiment demonstrates that the generative process successfully replaced the artificial markers with plausible environment details while leaving the rest of the scene mostly unaltered. This analysis is not perfect because it does not quantify, for example, the consistency of the shadows cast in the inpainted area, but it is sufficient for evaluating the consistency of the scene and the global illumination.

### 3.2. Saliency Maps: Experiments

For the qualitative analysis, inspired by the domain of interpretable AI, we sought to interpret and extract information about how the network learns. This is a fundamental phase in order to assess the capabilities of 6-DoF methods in other real-world scenarios, since metrics are limited in providing insight into a network’s comprehension.

Saliency methods are mainly developed for multi-class classification problems [49], and are thus sometimes referred to as Class Activation Maps (CAM) methods. At inference time, classification models output a probability score for each possible class, and the highest-scoring class is selected as the predicted one. However, in this study, we focus on a regression problem; this is pertinent as the gradient-based methods that we plan to use, Guided Backpropagation [46] and Grad-CAM [47], suppress the negative part of the gradient since it corresponds to a decrease in the score for the class of interest. In a scenario similar to ours (i.e, regression of rotation values in [−π,π]), we are clearly interested only in the magnitude of the gradient. Hereafter, we formalize the exact formulation of the saliency methods used in our work.

#### 3.2.1. Guided Backpropagation

It is straightforward to adapt the vanilla saliency for the regression. The rotation head of EfficientPose outputs a tensor $\bar{R}\in R^{N\times 3}$ of *N* candidate regressions. In a single-object scenario, as in our case, the rotation vector r for the target object is recovered as the one with the highest confidence. An identical approach can also be adopted for the translation regression. The gradient of r computed with respect to the input image *I* is a tensor with the same shape as the input image, which is reduced to a single channel by averaging. Unlike the original formulation, we retain both the negative and positive values of the gradient for the reasons mentioned above. For visualization, the saliency map (single-channel) is normalized to the interval [0,255] using min-max.

#### 3.2.2. Grad-CAM

**Figure 7:**
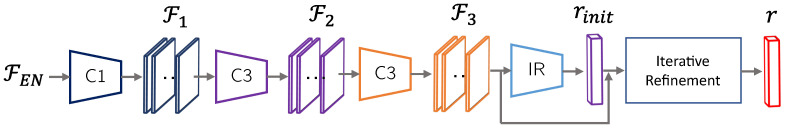
Rotation subnetwork for EfficientPose. The Translation subnetworks share a similar structure.

Adapting Grad-CAM to our problem is far more challenging. The original formulation operates on the feature map produced by the last convolutional layer of a classification model; however, the structure of the regression head of EP (simplified in Figure 7) makes it difficult to choose the correct feature map. Therefore, we opted to use a feature map obtained as the combination of the three convolutional layers that precede the output of the initial prediction ${\bar{r}}_{init}$, as the refinement module is used to predict small additive offsets to the initial prediction, which may be Identity mappings if the initial prediction does not require refinement.

Let $F_{1}$, $F_{2}$, and $F_{3}\in R^{\bar{W}\times \bar{H}\times \bar{C}}$, with $\bar{W},\bar{H},\bar{C}\in N$ be the three intermediate feature maps, we construct an aggregated feature map $F_{t}\in R^{\bar{W}\times \bar{H}\times 3\bar{C}}=\left\{F_{1}⊕F_{2}⊕F_{3}\right\}$ where ⊕ denotes the concatenation on the channel axis. Equation (2) is adapted by replacing $A^{k}$ with $F_{t}$ and replacing the average pooling with $L_{2}$ norm, in order to avoid opposing gradient values to elide each other in the sum. The new formulation for the pooled gradient becomes:

(4) $$\alpha_{t}=\sqrt{\sum_{i=1}^{\bar{W}}\sum_{j=1}^{\bar{H}}{\left(\frac{\partial r}{\partial F_{t}^{ij}}\right)}^{2}}\in R^{3\bar{C}}$$

The computation of the saliency maps is obtained by weighting each channel of $F_{t}$ with the corresponding value of $\alpha_{k}$ and accumulating over the channel axis to obtain a single matrix. Different from Equation (3), the ReLU is removed, and the absolute value of the weighted feature map is instead taken:

(5) $$L_{GC}=\sum_{t=1}^{3\bar{C}}\left|a_{t}F_{t}\right|$$

Replacing average pooling with an $L_{2}$ prevents cancellation between positive and negative gradients, but removes sign information. Unlike classification, where Grad-CAM encodes positive evidence for a class, our regression setting lacks a meaningful notion of positive versus negative evidence. Accordingly, there is no need to suppress negative evidence; hence, ReLU is replaced by absolute value. The resulting map reflects the magnitude of sensitivity of the output to spatial features. The use of absolute activation weighting is consistent with this interpretation, highlighting regions that most strongly influence the pose estimate irrespective of direction. The resulting saliency map is normalized and interpolated to the input image shape for visualization. These simple yet principled modifications result in a natural and effective generalization of Grad-CAM to the regression setting.

## 4. Results and Discussion

### 4.1. Experimental Setup

In this section, we present both qualitative and quantitative experiments to investigate the impact of ArUco markers on EfficientPose performance. In particular, we aim to address the following questions:

- Do ArUco markers in the LineMod dataset influence the predictions of EfficientPose, and can generative inpainting techniques mitigate this effect?
- What is the impact of removing ArUco markers only at test time on a model trained with marker-containing data?
- Can training on an ArUco-Free variant of the dataset recover the performance degradation?

#### 4.1.1. Data Preparation

To this end, we consider two variants of the LineMod dataset and train the standard version of EP (ϕ=0). Since the ArUco-Free Linemod (AF-LM) images cover only a subset of the original Linemod (LM) dataset (see Table 2), we restrict the LineMod data to the same subset to ensure a fair comparison. To keep the analysis concise, we focus on three sample objects: object 1 (the Ape), object 4 (the camera), and object 8 (the drill). We use the train/test split sizes reported in Table 2. For the quantitative evaluations (presented in Section 4.3), we employ stratified *k*-fold cross-validation (k=3) to mitigate the effects of the limited sample size.

#### 4.1.2. Saliency Analysis

We quantify the spatial distribution of model attention using the region-wise normalized saliency derived from Grad-CAM. Given a saliency map $S\in R^{H\times W}$ and a set of mutually exclusive binary masks corresponding to the object $\left(M_{\mathrm{obj}}\right)$, the ArUco marker $\left(M_{\mathrm{aruco}}\right)$, the board $\left(M_{\mathrm{brd}}\right)$, and the remaining pixels $\left(M_{\mathrm{other}}\right)$, we compute for each region *r* the mean saliency intensity as

(6) $$\mu_{r}=\frac{1}{|M_{r}|}\sum_{\left(i,j\right)\in M_{r}}S_{ij}$$

where $|M_{r}|$ denotes the number of pixels in region *r*. To enable comparison across regions and images, these values are further normalized to obtain relative attention scores

(7) $${\hat{\mu }}_{r}=\frac{\mu_{r}}{\sum_{k}\mu_{k}}\;k\in \mathrm{obj},\mathrm{aruco},\mathrm{brd},\mathrm{other}$$

Unlike the raw saliency mass, which is biased by region size, this formulation measures the average saliency per pixel, thereby capturing the intensity of attention independently of spatial extent. The resulting normalized scores ${\hat{\mu }}_{r}$ provide a decomposition of model attention across regions, allowing us to assess whether the model preferentially focuses on the object of interest, the ArUco marker, the board, or background region.

#### 4.1.3. Task Metrics

For the quantitative analysis of EfficientPose performance, we assess the standard literature metrics for 6D pose estimation tasks. In particular, the Average Distance (ADD) metric measures the mean distance between model points transformed by the estimated and ground-truth poses. Let M be the set of 3D model points, $\hat{P}=\left[R_{\mathrm{est}}|t_{\mathrm{est}}\right]$ the estimated pose, and $\bar{P}=\left[R_{\mathrm{gt}}|t_{\mathrm{gt}}\right]$ the ground-truth pose. The ADD error is defined as:

(8) $$\mathrm{ADD}=\frac{1}{\left|M\right|}\sum_{x\in M}{∥\hat{P}x-\bar{P}x∥}_{2}$$

For models with strong symmetries (not considered in this work), the symmetric ADD variant ADD-S is typically preferred.

In addition, we report the Mean Translation Error (MTE) and Mean Rotation Error (MRE). The MTE, expressed in millimeters (mm), specifically isolates the Euclidean distance between the estimated translation vector $t_{est}$ and the ground truth $t_{gt}$ in the 3D space. This metric quantifies the global spatial drift independently of any rotational bias. Conversely, the MRE, expressed in degrees (deg), expresses the average geodesic distance between the estimated and reference rotation matrices. Formally, given the rotation matrices $R_{est}$ and $R_{gt}$, the error is derived from the trace of the relative rotation matrix as follows:

(9) $$\Delta \theta =\arccos\left(\frac{\mathrm{Tr}(R_{est}R_{gt}^{-1})-1}{2}\right)$$

The combined use of ADD, MTE, and MRE ensures a high-granularity analysis, distinguishing between errors caused by camera-to-object distance (translation) and those related to the object’s orientation in the scene (rotation).

### 4.2. Qualitative Analysis

#### 4.2.1. Markers Distribution

**Figure 8:**
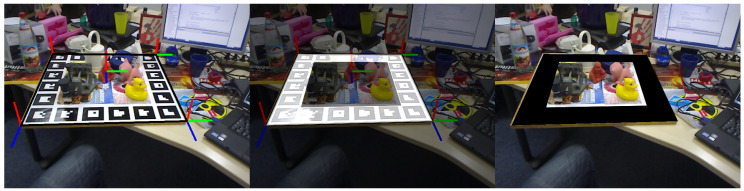
Visualization of the geometric procedure used to localize ArUco markers in the scene.

We qualitatively analyze the spatial distribution of ArUco markers within the images. We leverage the available ground-truth object poses to estimate the spatial location of the planar support containing the ArUco markers. Assuming the object reference frame origin at [0,0,0], the four corners of the marker plane can be defined in 3D as fixed offsets $\left[\pm \delta_{x},\pm \delta_{y},\pm \delta_{z}\right]$. These correspond to the corners of the underlying chessboard structure on which the objects are placed. For each image, the known rotation and translation are applied to project these 3D points onto the image plane, enabling a consistent localization of the marker regions (see Figure 8). This process is used solely for visualization and analysis purposes.

**Figure 9:**
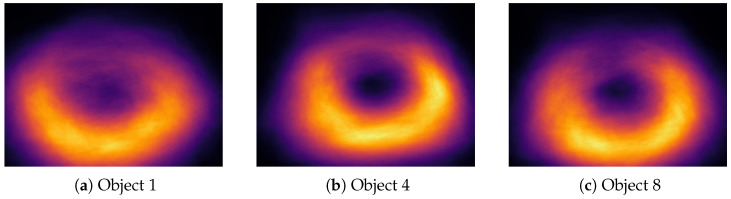
Distribution of the ArUco Board in LineMod Images for different object subsets.

The resulting ArUco masks are employed to compute density maps that highlight the spatial distribution of markers across the dataset. The results in Figure 9 show that markers are not uniformly distributed; instead, they tend to concentrate in specific regions of the images. This preliminary analysis provides insight into dataset-specific biases, revealing that ArUco markers are consistently localized in restricted regions rather than uniformly distributed across the image plane.

#### 4.2.2. Saliency Maps Analysis

##### Guided Backpropagation on LineMod

**Figure 10:**
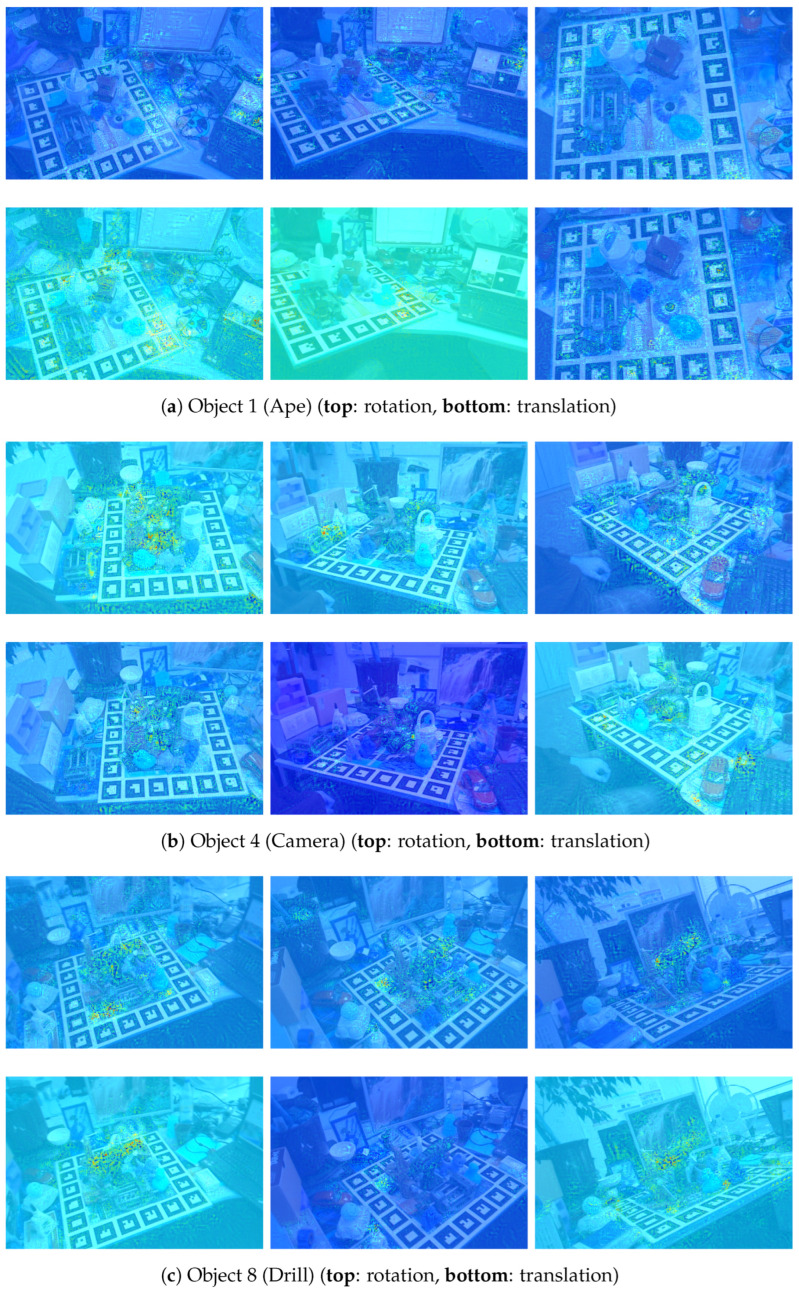
Saliency maps for the EfficientPose model trained on **LineMod**. Rotation and translation sub-task. Obtained using the **Guided Backpropagation** method.

To begin, we analyze saliency maps obtained using the simplest method: guided backpropagation. This choice allows us to establish a foundation for qualitative observations; unlike the more advanced Grad-CAM, extending this method to regression tasks is more straightforward, although the resulting maps are sparser and less informative. First, we visualize the saliency maps on the original LM dataset, using the EP weights provided by the authors (https://github.com/ybkscht/EfficientPose, accessed on 15 November 2025) to ensure an unbiased initial evaluation. We compute the saliency maps with respect to both the rotation and translation estimation sub-tasks. In Figure 10, we show a few indicative sample saliency maps for three distinct Linemod objects: object 1 (the Ape), object 4 (the camera), and object 8 (the Driller). These have been selected to illustrate some of the patterns that emerge.

Generally speaking, we can observe three distinct phenomena:

- Object 1 shows a significant level of saliency activation concentrated around the ArUco markers, while the target object (the Ape) is seldom activated.
- For object 4, we observe a mixed saliency activation between the target object (the camera) and the surrounding area, including the marker board.
- For object 8, we observe significant saliency activation on the target object (the drill), although a degree of saliency that affects the object’s surroundings—including the grid of markers—still appears evident.

In all cases, the sparsity of the maps obtained using guided backpropagation does not allow for the identification of significant differences between the rotation and translation subtasks.

##### Generalized GradCAM on LineMod

**Figure 11:**
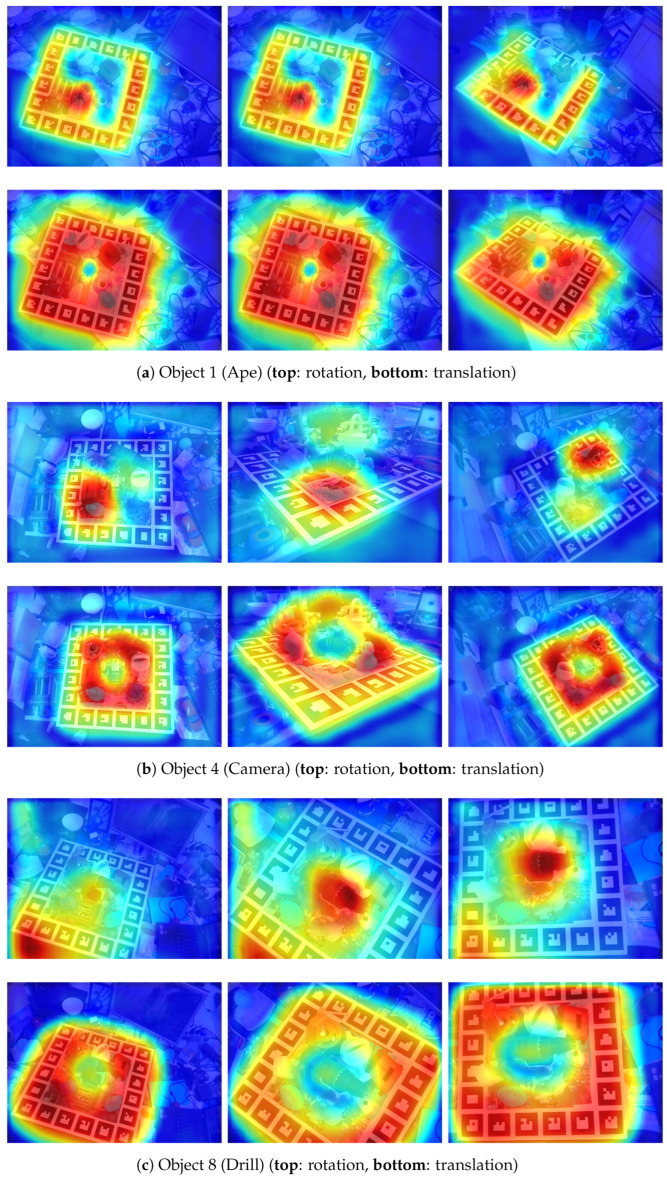
Saliency maps for the EfficientPose model trained on **LineMod**. Rotation and translation sub-task. Obtained using the **Generalized GradCAM** method.

Below, we extend the analysis presented earlier using the GradCAM method generalized for regression tasks introduced in Section 3.2. While vanilla backpropagation relies on raw gradients that tend to be noisy and less semantically meaningful, Grad-CAM captures high-level semantic regions by leveraging feature maps from deeper layers, leading to more stable and human-interpretable visual explanations. By analyzing the saliency maps obtained for the rotation and translation subtasks, respectively, a sample of which is shown in Figure 11, we can gain stronger insight into the EfficientPose behavior when trained on LineMod.

A consistent pattern emerges for the translation subtask: across all considered objects, saliency is strongly concentrated over the marker grid. This suggests that the model may rely, at least in part, on structured background cues when estimating translation, rather than exclusively on object-specific features. For the rotation subtask, the behavior appears more heterogeneous and aligns with the observations from the guided backpropagation maps:

- For Object 1 (Ape), the markers contribute significantly to the prediction, and a notable response is also observed on a neighboring object (Bench Vise), indicating sensitivity to contextual elements.
- For Object 4 (Camera), saliency is predominantly located on a group of surrounding objects rather than on the target itself, suggesting that contextual cues may play a dominant role in this case.
- For Object 8 (Driller), saliency is largely concentrated on the target object, although nearby regions still exhibit non-negligible influence.

To complement the qualitative observations, we quantify the distribution of the saliency score with the methodology introduced in Section 4.1.2. The results are reported in Table 3 for the model trained on the LineMod images. These results are consistent with the qualitative observations: the translation subtask shows the highest density of localized saliency in the marker region, especially for objects 1 and 8. For object 4, we observe a concentration of saliency in the board region, suggesting the use of surrounding objects as a reference. Overall, these observations are consistent with the presence of dataset-induced biases, where structured elements such as the marker grid or neighboring objects can influence the model predictions. However, the extent and nature of this dependence vary across objects and subtasks, indicating that the underlying mechanisms are non-trivial.

##### Generalized GradCAM on ArUco-Free LineMod

**Figure 12:**
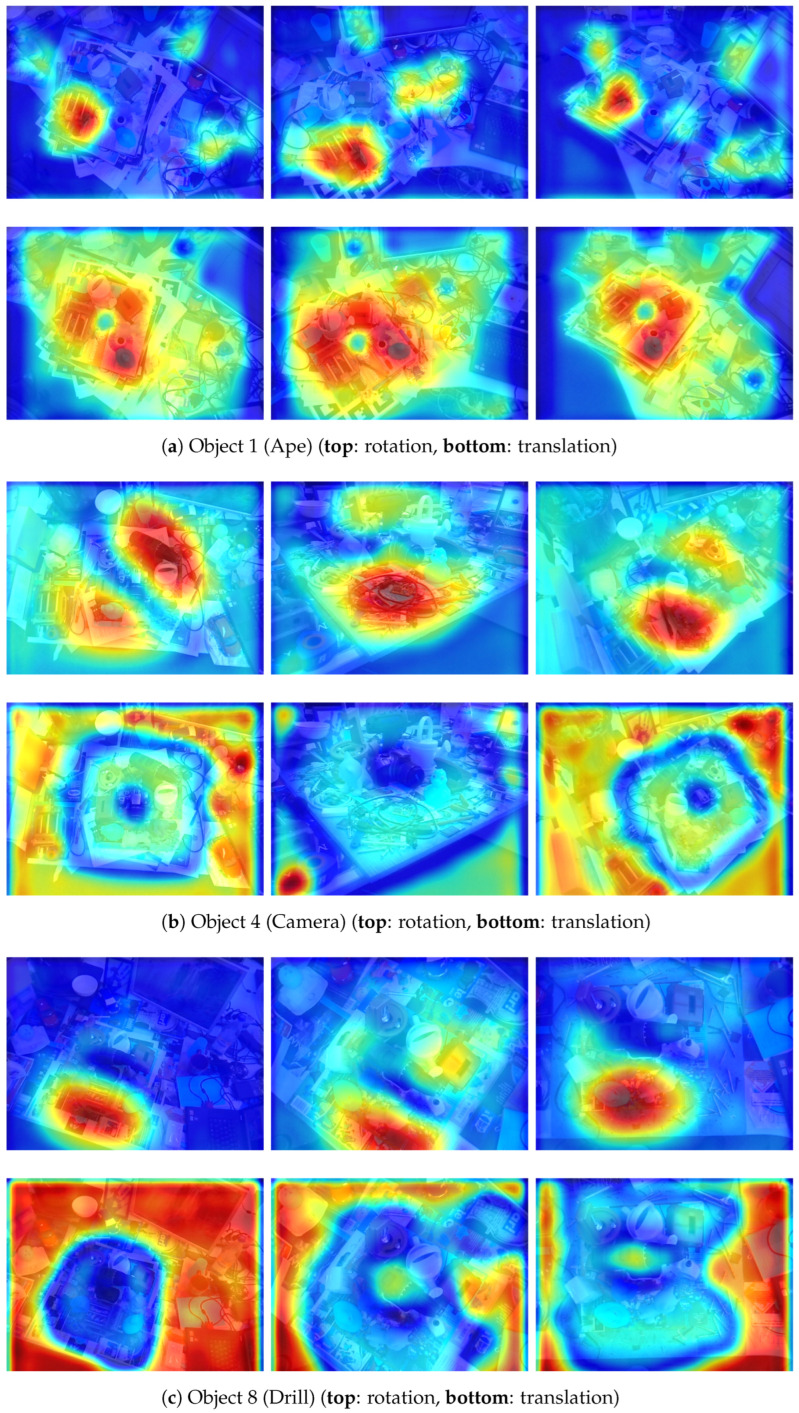
Saliency maps for the EfficientPose model trained on **ArUco-Free LineMod**. Rotation and translation sub-task. Obtained using the **Generalized GradCAM** method.

To conclude the qualitative analysis, we analyze the saliency maps obtained for the EfficietPose model retrained on the proposed ArUco-Free dataset introduced in this work. Object 1 shows the most obvious dependence on ArUco markers in the LineMod settings. In the ArUco-Free setting Figure 12a, the radical change is particularly noteworthy, in particular for the rotation sub-task. The saliency density in the region formerly occupied by the ArUco markers is significantly reduced, though there is a greater focus on the background area. For object 4 (the camera), we observe a strong reliance on the surrounding objects for the rotation sub-tasks, similarly to what was observed in the original setup. The most striking difference is observed in the translation subtask: while with LineMod the strong reliance on visual cues from the marker board area is evident, the ArUco-Free dataset eliminates this shortcut. However, the model’s focus is still clearly linked to a great extent to spatial clues. To complement the qualitative analysis of saliency maps, we extend the analysis of the saliency distribution to the ArUco-Free setting and report the results in Table 4. The results confirm a general reduction in saliency in the marker region, but also indicate a shift in focus toward the board and background regions.

##### Discussion

These observations suggest that, while generative inpainting mitigates the dependence on explicit marker cues, it does not entirely prevent the use of contextual information. The model appears to adapt by leveraging alternative spatial patterns, suggesting that background-induced biases are only partially addressed. This is likely due to the intrinsic nature of the data acquisition process: since the scenes are captured by a moving camera, the background and surrounding elements move rigidly and synchronously with the target object. Consequently, the model adapts by leveraging these alternative spatial patterns, suggesting that background-induced biases are only partially addressed through inpainting alone. The next section provides a quantitative analysis to further investigate these observations.

### 4.3. Quantitative Analysis

Before presenting the results, we clarify our evaluation protocol. While most experiments follow a standard train/test split to manage the significant computational cost of training, we selected Object 4 (Camera) as a representative case study for a more detailed statistical validation. For this object, we performed a 3-fold cross-validation across the entire available subset, as shown in Table 5. This allows us to report the results as Mean ± Standard Deviation (σ), providing an estimate of the model’s stability against different data distributions without the need for exhaustive re-training of every object class. For the other objects (Ape and Driller), we report results from a single split, as their performance trends consistently align with the behavioral patterns observed and validated in the Camera case study.

#### 4.3.1. Object 1: The Ape

The performance of EfficientPose for the *Ape* object (Object 1) is reported in Table 6. Consistent with the saliency map analysis, this object exhibits a strong dependency on the ArUco marker region. The best performance is achieved when the model is both trained and evaluated on the original LineMod (LM) dataset, yielding the highest ADD score (0.88) and the lowest translation and rotation errors (MTE: 5.42 mm, MRE: 3.96°). However, this performance does not generalize across domains. When the same model is evaluated on the ArUco-Free (AF-LM) dataset, performance drops dramatically (ADD: 0.15, MTE: 22.76 mm, MRE: 16.17°), confirming that the model has learned to rely heavily on the presence of markers as a shortcut for pose estimation. Training on the AF-LM dataset leads to more consistent results across both test sets, with identical ADD scores (0.80) and reduced performance gaps. Nevertheless, this comes at the cost of lower peak performance compared to the LM-trained model. In particular, while the model becomes less sensitive to the presence or absence of markers, it underperforms the LineMod version, especially in terms of rotation (MRE).

#### 4.3.2. Object 4: The Camera

**Figure 13:**
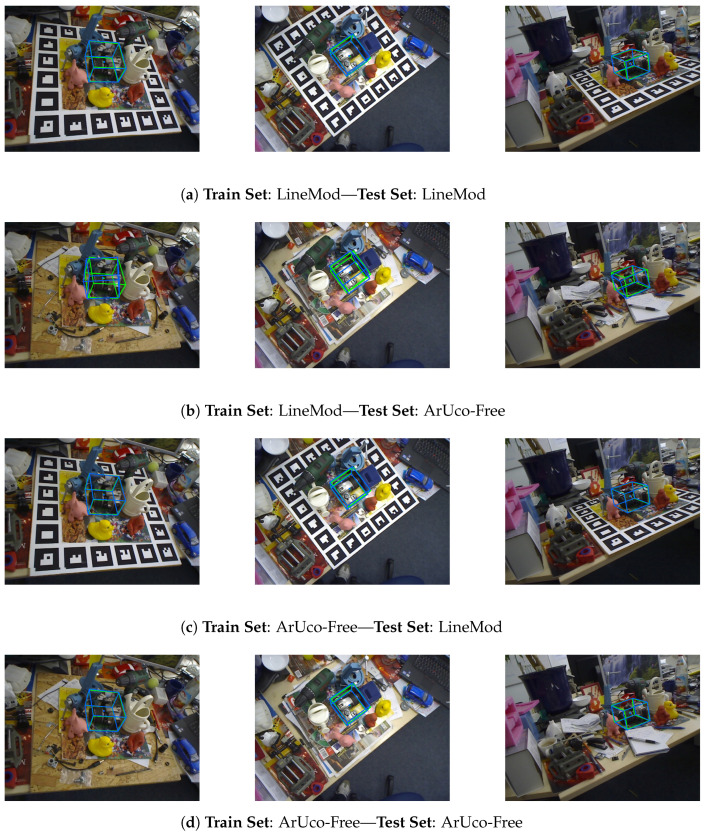
Qualitative results on Object 4 using EfficientPose (ϕ=0). The green and blue 3D bounding boxes represent the ground-truth and estimated poses, respectively. The rows illustrate different training and testing configurations: (**First row**) original LineMod training and testing; (**Second row**) original LineMod training tested on the ArUco-Free dataset; (**Third row**) training on the proposed ArUco-Free dataset tested on original LineMod; (**Fourth row**) both training and testing performed on the ArUco-Free dataset.

Table 5 summarizes the performance of EfficientPose for the “Camera” (Object 4). As mentioned, these values represent the average across the k-fold cross-validation. Initially, when the network is trained on the standard Linemod dataset and tested on the same domain, it achieves an ADD metric of 0.98±0.0026, with a Mean Translation Error (MTE) of 6.03±1.22 mm and a Mean Rotation Error (MRE) of 3.45°±0.42°. These low variance values indicate a high level of confidence in the original domain. However, a dramatic performance collapse and a significant increase in instability are observed when this same model is evaluated on the ArUco-Free Validation set. In this cross-domain scenario, the ADD accuracy drops to 0.39±0.0632, while the MTE and MRE increase to 20.03±21.93 mm and 12.23°±1.47°, respectively. The high standard deviation in translation error (σ=21.93 mm) highlights how the absence of markers leads to erratic behavior in depth estimation. As shown in the saliency maps and the visualizations in Figure 13, the domain shift from LineMod to ArUco-Free causes severe and inconsistent misalignments once artificial landmarks are removed. In contrast, the model trained on our ArUco-Free dataset demonstrates significantly higher robustness and remarkable stability. When validated on the ArUco-Free set, it maintains a stable ADD accuracy of 0.92±0.0090, with an MTE of 7.79±0.60 mm and an MRE of 4.14°±0.15°. The drastic reduction in standard deviation (from 21.93 to 0.60 mm for MTE) proves that the generative inpainting process successfully decoupled the network from marker-dependency, leading to a more generalized and reliable pose estimation. Notably, this model also generalizes effectively back to the original Linemod domain, achieving an ADD of 0.94±0.0094 and an MRE of 3.98°±0.32°. These quantitative results, supported by the bounding box comparisons in Figure 13, demonstrate that training on “cleaned” scenes provides superior generalization without sacrificing precision on the target objects.

#### 4.3.3. Object 8: The Driller

We repeat the same analysis for Object 8 (the Driller). We evaluate the performance of EfficientPose (ϕ=0) by comparing models trained on the **Original LM dataset** and our **ArUco-Free dataset**. The experimental results for this object, summarized in Table 7, are strictly in line with those observed for the “Camera” (Object 4). Specifically, when the model trained on the original dataset is tested on the ArUco-Free validation set, we observe a significant performance degradation: the ADD accuracy collapses and both the MTE and MRE increase, as reported in Table 7. Conversely, the model trained on our ArUco-Free dataset demonstrates superior generalization and stability. As shown in the comparative analysis, this model not only achieves high accuracy on the ArUco-Free test set but also maintains good performance when evaluated on the original Linemod dataset.

### 4.4. Discussion

The behavior observed in the qualitative analysis presented above clearly highlights a strong sensitivity to domain shift in models trained on LineMod, whose performance deteriorates when tested on the ArUco dataset. Conversely, models trained on the ArUco-Free dataset exhibit stronger generalization ability to cross-dataset background variations. In the first instance, these findings reinforce the idea that removing artificial markers through generative inpainting prevents the network from relying on localized, high-contrast features (such as the ArUco black borders). A detailed analysis of the saliency maps (see Figure 12) indicates that such an improvement is only partial. Generative inpainting diversifies the background, effectively introducing a powerful regularizing effect attributable to data augmentation and domain randomization methods. In practice, the model is tasked to extract features that are more robust to background variations; however, in the absence of an explicit constraint during the optimization phase, we cannot, in principle, conclude that the emphasis is placed solely on the target object.

## 5. Conclusions

In this study, we investigated shortcut learning in bottom-up 6-Degrees-of-Freedom object pose estimation models, which process the entire scene and may exploit contextual cues rather than object geometry. Using EfficientPose trained on the LineMod dataset, saliency map analysis (Section 4.2.2) revealed a strong reliance on ArUco markers, surrounding objects, and background, supporting the presence of shortcut biases. To further investigate this effect, we introduced an ArUco-Free version of LineMod obtained via generative inpainting. We observed mixed results: quantitative results (ADD, MTE, MRE) show that models trained on the original dataset fail to generalize to marker-free images, while training on the ArUco-Free dataset improves cross-domain robustness. However, saliency analysis indicates that models still partially rely on spatial context. This behavior is likely due to the dataset acquisition process, where the background and surrounding objects move rigidly with the target; the model may still exploit these consistent spatial correlations as alternative cues for pose estimation. Overall, our findings highlight that, while removing explicit markers effectively neutralizes explicit, high-contrast markers, the biases induced by scene structure and shared rigid-body motion between the target and its background remain. Addressing these factors is essential for developing more robust and generalizable pose estimation models.

### Future Work

The results of this study suggest numerous insights that may point to relevant research directions in the field of 6D pose estimation and, more generally, in understanding the shortcut bias present in benchmarking datasets. Our study focused exclusively on EfficientPose because it is a simple model based on a bottom-up approach; this type of model is still not widely used, partly due to the difficulty of finding suitable datasets. Our findings can support future research on this type of architecture. Furthermore, the proposed ArUco-Free dataset has proven to be a powerful tool for investigating the phenomenon of shortcut learning, but it does not provide a comprehensive solution to this problem. Future research will focus on addressing the other identified shortcuts and on achieving true generalization capabilities in real-world settings by leveraging photorealistic generation of the entire dataset [50]. The generalized GradCAM approach, together with the analysis framework introduced in Section 4.1.2, provides a solid foundation for interpreting model predictions. Extending this analysis through comparisons with explainability methods such as SHAP (SHapley Additive Explanations) [51] and LIME (Local Interpretable Model-agnostic Explanations) [52] could offer further insights into the decision-making mechanisms underlying these models.

## Figures and Tables

**Table 1:** Evaluation of mean pixel-wise difference for the ArUco-Free images with respect to the corresponding LineMod image.

Metric	Value
Total valid images processed	2301
avg. mean difference (all pixels)	19.11
avg. mean difference (ArUco pixels)	214.20
avg. mean difference (Non-ArUco pixels)	19.82

**Table 2:** Comparison between the original LineMod (LM) dataset and the ArUco-Free (AF) LineMod variant. Objects in **bold** are used for evaluation in this paper.

Obj	Name	LM Original			AF Linemod		
		Total	Train	Test	Total	Train	Test
**1**	**APE**	1236	186	1050	170	144	26
2	BENCHVS	1212	181	1031	170	144	26
**4**	**CAMERA**	1201	181	1020	464	180	284
5	CAN	1196	180	1061	170	144	26
**8**	**DRILLER**	1188	179	1009	212	180	32
9	DUCK	1254	189	1065	170	144	26
10	EGGBOX	1253	188	1065	170	144	26
11	GLUE	1220	184	1036	170	144	26
12	HOLEPN	1237	186	1051	170	144	26
13	IRON	1152	1148	979	170	144	26
14	LAMP	1227	185	1042	170	144	26
15	PHONE	1243	184	1041	137	144	26

**Table 3:** Distribution of saliency on the **LineMod** dataset. Saliency scores across regions for the three evaluated objects and the two considered sub-tasks. For each setting, we report the normalized mean saliency assigned to the object, the ArUco marker, the background, and the remaining pixels.

Object	Sub-Task	Object	Markers	Board	Other
Object 1	Rotation	**0.31**	0.28	0.28	0.13
	Translation	0.17	**0.35**	0.33	0.14
Object 4	Rotation	**0.28**	0.24	0.31	0.18
	Translation	0.23	0.27	**0.35**	0.14
Object 8	Rotation	**0.30**	0.24	0.28	0.18
	Translation	0.21	**0.35**	0.31	0.14

**Table 4:** Distribution of saliency on the **ArUco-Free** dataset. (*) The column “Markers” refers to the region where markers are present in the original LineMod images, even though they have been removed in this ArUco-Free version.

Object	Task	Object	Markers *	Board	Other
Object 1	Rotation	**0.31**	0.22	0.29	0.18
	Translation	0.22	0.26	**0.34**	0.18
Object 4	Rotation	0.23	0.26	**0.31**	0.19
	Translation	0.18	0.25	**0.29**	**0.29**
Object 8	Rotation	0.26	0.24	**0.33**	0.16
	Translation	0.18	0.24	0.20	**0.38**

**Table 5:** EfficientPose results for **Object 4** (the Camera). ADD is computed on the two datasets with the two sets of trained parameters available (trained with ϕ=0). Then, in the right column, for each training, the two test results’ averages are computed, in order to observe which experiment performs better both on the test images of the same image type seen during training, and on the test images of the type never seen. The reported values represent the Mean ± Standard Deviation (σ) calculated across the three runs of the k-fold cross-validation.

Dataset Mode	ADD (↑)		MTE (mm) (↓)		MRE (deg) (↓)	
	LM	AF-LM	LM	AF-LM	LM	AF-LM
LM	0.98_±0.0026_	0.39_±0.0632_	6.03_±1.22_	20.03_±21.93_	3.45_±0.42_	12.23_±1.47_
AF-LM	0.94_±0.0094_	0.92_±0.0090_	6.95_±0.4155_	7.79_±0.60_	3.98_±0.32_	4.14_±0.15_

**Table 6:** EfficientPose results for **Object 1** (the Ape). ADD is computed on the two datasets with the two sets of trained parameters available (trained with ϕ=0). Then, in the right column, for each training, the two test results’ averages are computed, in order to observe which experiment performs better both on the test images of the same image type seen during training, and on the test images of the type never seen.

Dataset Mode	ADD (↑)		MTE (mm) (↓)		MRE (deg) (↓)	
	LM	AF-LM	LM	AF-LM	LM	AF-LM
LM	**0.88**	0.15	**5.42**	22.76	**3.96**	16.17
AF-LM	0.80	0.80	8.14	17.70	16.10	13.32

**Table 7:** EfficientPose results for Object 8 (Driller). ADD is computed on the two datasets with the two sets of trained parameters available (trained with ϕ=0). Then, in the right column, for each training, the averages of the two test results are computed, in order to observe which experiment performs better both on the test images of the same type of the images seen during training, and on the test images of the type never seen.

Dataset Mode	ADD (↑)		MTE (mm) (↓)		MRE (deg) (↓)	
	LM	AF-LM	LM	AF-LM	LM	AF-LM
LM	0.99	0.59	6.45	22.11	4.22	14.35
AF-LM	0.81	1.00	14.35	13.08	9.21	7.93

## Data Availability

The dataset proposed in this study is openly available at the following repository: https://github.com/cscribano/GEN_AF_LineMod (accessed on 26 May 2026).
